# Systematic Quality Improvement in Dobutamine Stress Echocardiography: A Two-Cycle Audit Demonstrating Enhanced Diagnostic Accuracy

**DOI:** 10.7759/cureus.100628

**Published:** 2026-01-02

**Authors:** Milan Mehta, Nileshkumar Khanpara

**Affiliations:** 1 Cardiology, Sardar Patel Hospital and Heart Institute, Ankleshwar, IND; 2 General Medicine, Om Hospital and ICU, Surat, IND

**Keywords:** clinical audit, coronary angiography, coronary artery disease, dobutamine stress echocardiography, false negatives, false positives, medication verification checklist, non-invasive cardiology, patient safety, quality improvement

## Abstract

Background: Dobutamine stress echocardiography (DSE) has become an important and frequently used non-invasive diagnostic tool for assessing significant coronary artery disease (CAD) in clinical practice. This technique offers several advantages over alternative diagnostic methods, including the absence of radiation exposure, relatively widespread availability across healthcare facilities, and cost-effectiveness compared to nuclear imaging or cardiac magnetic resonance imaging. However, the diagnostic accuracy of DSE depends heavily on multiple factors, including the quality of image acquisition, appropriate patient preparation, and the interpretative expertise of the performing physician.

As an operator-dependent investigation, DSE requires continuous quality assessment to maintain diagnostic accuracy and ensure optimal patient outcomes. Without systematic monitoring, variations in technique, interpretation, and patient preparation can lead to suboptimal diagnostic performance, potentially resulting in false negative results that miss significant coronary disease or false positive findings that lead to unnecessary invasive procedures. Systematic audit provides a structured framework for identifying performance gaps, comparing results against established benchmarks, and implementing evidence-based improvements.

Regular audit cycles enable clinicians to objectively evaluate their diagnostic accuracy against the reference standard of coronary angiography (CAG), identify specific areas requiring improvement, and implement targeted corrective measures. This approach ensures that patients and referring physicians can have confidence in the diagnostic reliability of the DSE test while promoting continuous professional development and maintaining high clinical standards.

Objectives: To evaluate DSE diagnostic accuracy against CAG and assess improvement following specific quality interventions. This two-cycle audit applied the Plan-Do-Study-Act (PDSA) framework and assessed diagnostic performance using sensitivity, specificity, positive predictive value (PPV), negative predictive value (NPV), and overall accuracy measured against CAG as the reference standard.

Methods: Two-cycle clinical audit conducted in a non-invasive cardiology practice. Cycle 1 (October 2021-September 2024) included 160 DSE procedures; 75 (46.9%) underwent CAG procedures. Following analysis and intervention implementation, Cycle 2 (February 2025-October 2025) included 27 DSE procedures with 18 (66.7%) subsequent CAG procedures. The primary outcome was concordance between DSE and CAG results. The sensitivity, specificity, PPV, NPV, and overall accuracy were calculated.

Results: Overall accuracy improved from 82.7% (62/75) in Cycle 1 to 88.9% (16/18) in Cycle 2, representing a 6.2 percentage point improvement. False positive results were eliminated entirely (4.0% to 0%), while false negatives decreased modestly (13.3% to 11.1%). Analysis revealed that both false negatives in Cycle 2 resulted from failure to withhold nitrate therapy, prompting implementation of a three-tier medication verification checklist.

Conclusions: Systematic audit with targeted interventions successfully enhanced DSE diagnostic accuracy. The medication verification checklist addresses a preventable systematic error and represents a transferable safety strategy applicable to other stress testing modalities.

## Introduction

Dobutamine stress echocardiography (DSE) serves as a cornerstone non-invasive modality to assess risk for coronary artery disease (CAD), offering advantages including absence of radiation exposure, widespread availability, and cost‑effectiveness [1-3]. However, diagnostic accuracy depends heavily on technical image quality, appropriate patient preparation, and interpretative expertise. Like all operator‑dependent investigations, DSE requires ongoing quality assurance to maintain performance standards and optimize patient outcomes [4-6].

Published guidelines from the American Society of Echocardiography and the European Association of Echocardiography emphasize the importance of structured quality assurance programs in stress echocardiography laboratories [7-10]. Clinical audit provides a framework for measuring performance against benchmarks, identifying gaps, and implementing targeted improvements [8].

## Materials and methods

This quality improvement project was conducted as a two‑cycle clinical audit utilizing the Plan‑Do‑Study‑Act (PDSA) framework. All patients undergoing DSE in a single non‑invasive cardiology practice were eligible for inclusion. For the purposes of diagnostic concordance analysis, only those patients who subsequently underwent coronary angiography (CAG) were considered. Significant CAD was defined as a ≥70% diameter stenosis in a major epicardial coronary vessel or a ≥50% stenosis in the left main coronary artery, as determined by quantitative CAG [1]. Dobutamine stress testing followed the standard incremental protocol (5-40 μg/kg/min), with atropine (up to 1.2 mg) administered when the target heart rate was not achieved. Wall-motion assessment adhered to the American Society of Echocardiography's 17-segment model, and DSE was considered positive when new or worsening wall-motion abnormalities occurred in at least two contiguous segments. These technical standards were consistently applied in both cycles to ensure reproducibility.

The accepted benchmarks for DSE in detecting significant CAD are an overall diagnostic accuracy exceeding 80%, sensitivity greater than 80%, and specificity above 85% [2-6].

In the first audit cycle, conducted between October 2021 and September 2024, a total of 160 DSE procedures were performed, of which 75 patients (46.9%) subsequently underwent CAG. Concordance between DSE and CAG was observed in 62 cases, yielding an overall diagnostic accuracy of 82.7% (62/75). Discordant findings included 10 false negatives (13.3%) and three false positives (4.0%).

Detailed review of the false-negative cases revealed several contributing factors: submaximal stress achievement, technical limitations in image acquisition, single‑vessel disease with collateralization, and disease progression occurring between the DSE and CAG examinations [2,3,7]. The false-positive results were attributed to overinterpretation of subtle wall motion abnormalities and the presence of microvascular dysfunction [4,5].

Following the baseline audit, several targeted quality improvement measures were introduced to enhance the diagnostic performance of DSE. First, standard imaging protocol's strict adherence was enforced through, first, standardized acquisition techniques emphasizing optimal endocardial border visualization [1,4]. Second, a structured reporting template was mandated to ensure consistent documentation of stress adequacy, coronary territory assessment, image quality, and diagnostic classification. Third, a program of regular peer review was established, involving monthly evaluation of all cases with subsequent angiography, with particular focus on discordant findings [8]. Fourth, patient selection criteria were updated to improve assessment of pre‑test probability and referral appropriateness [6,7]. Each intervention directly targeted performance gaps identified in Cycle 1, specifically variation in acquisition techniques, inconsistent documentation, limited peer-review oversight, and inadequate medication-interference verification, ensuring that each action addressed a defined deficiency. Finally, continuing medical education was reinforced through focused workshops addressing image acquisition and interpretation pitfalls [9-12].

Cycle 2 was conducted between February 2025 and October 2025, during which 27 consecutive DSE procedures were performed following implementation of the quality improvement interventions. Of these, 18 patients (66.7%) subsequently underwent CAG, forming the analysis cohort. To ensure validity and comparability with Cycle 1, the same inclusion criteria, standardized DSE protocol, CAG reference standard, and data collection methodology were applied. Diagnostic accuracy metrics (sensitivity, specificity, positive predictive value (PPV), negative predictive value (NPV), and overall accuracy) were calculated for both cycles [6,13-16]. Improvements were quantified by percentage point differences between cycles, and all false‑positive and false‑negative cases in Cycle 2 were subjected to detailed review to identify contributing factors and guide further quality improvement [8].

## Results

Overall diagnostic accuracy improved from 82.7% (62/75) in Cycle 1 to 88.9% (16/18) in Cycle 2, representing a 6.2 percentage point gain following implementation of targeted quality improvement measures. Importantly, false-positive results, which accounted for 4.0% (3/75) of cases in Cycle 1, were completely eliminated in Cycle 2. This finding underscores the effectiveness of interventions such as enhanced imaging protocols, structured reporting, and peer review in improving interpretative specificity and reducing unnecessary invasive procedures.

False-negative results decreased modestly, from 13.3% (10/75) in Cycle 1 to 11.1% (2/18) in Cycle 2. Although this reduction was relatively small, the identification of a clear and preventable cause, failure to withhold nitrate therapy, highlighted a critical process gap. The subsequent introduction of a three‑tier medication verification checklist directly addressed this issue, establishing a reproducible safeguard against pharmacological interference with test sensitivity.

**Figure 1:**
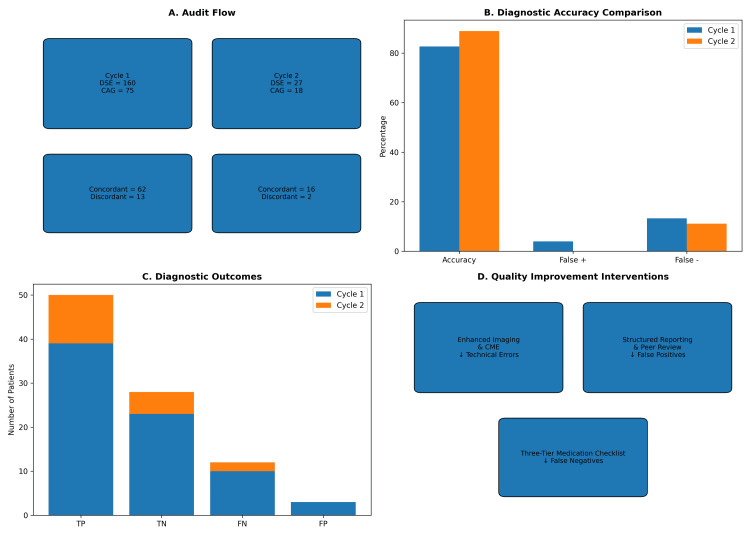
Summary of Diagnostic Audit Findings and Quality Improvement Interventions Across Two Cycles Panel A illustrates the audit flow, showing the number of dobutamine stress echocardiography (DSE) procedures and subsequent coronary angiographies (CAG) in Cycle 1 and Cycle 2, along with concordant and discordant diagnostic outcomes. Panel B compares diagnostic accuracy metrics between cycles, highlighting improvements in overall accuracy and reductions in false-positive and false-negative rates. Panel C presents the distribution of diagnostic outcomes (true positives (TP), true negatives (TN), false negatives (FN), and false positives (FP)) for each cycle. Panel D summarizes the targeted quality improvement interventions implemented between cycles, linking each strategy to its corresponding impact on diagnostic performance. CME: continuing medical education

Figure 1 summarizes the audit flow, diagnostic performance metrics, and quality improvement interventions implemented across both cycles. Panel A outlines the number of DSE procedures and subsequent CAGs, along with concordant and discordant outcomes. Panels B and C highlight improvements in diagnostic accuracy and distribution of true and false diagnostic outcomes. Panel D links each intervention to its corresponding impact, illustrating how enhanced imaging, structured reporting, and medication verification contributed to reduced error rates.

In addition, the proportion of patients proceeding to CAG increased substantially, from 46.9% (75/160) in Cycle 1 to 66.7% (18/27) in Cycle 2. This rise may reflect improved patient selection, with DSE being more consistently applied to individuals at a higher pre‑test probability of disease. While higher angiography rates can increase verification bias, they also strengthen confidence in the observed concordance by ensuring that diagnostic comparisons are made in clinically relevant populations.

As CAG was performed only in a subset of patients, undetected false-negative results among patients who did not undergo angiography cannot be conclusively excluded.

A structured three‑tier medication verification checklist was introduced in Cycle 2, requiring independent confirmation by nursing staff, cardiac technicians, and the performing physician before test initiation. The complete checklist form, outlining each verification step and signature requirement, is provided in the Appendices to facilitate replication and adaptation in other clinical settings.

## Discussion

This two‑cycle audit demonstrates that systematic quality improvement interventions can meaningfully enhance the diagnostic accuracy of DSE [8,15]. The identification of medication‑related false negatives highlighted a preventable system‑level error, reinforcing the importance of robust procedural safeguards in stress testing protocols [2]. The most notable improvement was the complete elimination of false-positive results in Cycle 2, reflecting enhanced specificity and more consistent interpretation of wall motion abnormalities [9,11]. Although the reduction in false negatives was modest, the recognition that both cases were linked to nitrate continuation underscores the critical role of medication management in optimizing test sensitivity [2]. In addition, the higher rate of CAG observed in Cycle 2 may indicate improved patient selection, with DSE being more consistently applied to individuals at a greater pre‑test probability of disease [6,7].

The strengths of this audit include systematic data collection, adherence to established diagnostic standards, and the structured application of the PDSA framework [1,6,15]. Limitations include the relatively small sample size in Cycle 2, the retrospective design, potential verification bias, and the single‑center setting, which may limit generalizability. Functional versus anatomical testing comparisons further contextualize the diagnostic role of DSE [16].

Based on these findings, several recommendations are proposed. Structured reporting, regular peer review, and continuing medical education should be maintained to ensure consistency and interpretative accuracy [15]. Importantly, previous quality improvement initiatives have demonstrated that embedding audit cycles into routine practice can significantly reduce inappropriate or discordant echocardiographic studies [12]. Building on this evidence, continued reinforcement of structured reporting and peer review is advised [13-15]. Institutional protocols should be developed to guide appropriate referral for DSE and subsequent CAG [6,7]. Regular multidisciplinary review of discordant cases is recommended to foster collective learning and improve diagnostic reliability [17,18]. Universal adoption of the three‑tier medication verification checklist is strongly encouraged as a transferable safety intervention to prevent drug‑related diagnostic interference [2,17]. Finally, a third audit cycle should be planned to evaluate the sustainability of these improvements and to formally assess the effectiveness of the checklist intervention [8].

It must be reiterated that this audit has several limitations that warrant consideration. The relatively small sample size in Cycle 2 reduces statistical power and limits the generalizability of findings; however, quality improvement projects are primarily designed to evaluate process changes rather than achieve population‑level statistical significance [8,15]. The single‑center design may introduce institutional bias, yet it also allowed for consistent application of interventions and close monitoring of outcomes [6,7]. Verification bias is possible given that only patients proceeding to CAG were included in concordance analysis, but this approach ensured comparison against the accepted gold standard [1,16]. Despite these constraints, the audit demonstrated measurable improvements in diagnostic accuracy and eliminated false-positive results, underscoring the practical value of systematic interventions [9,11,19]. Future directions include expanding the audit to multiple centers, increasing sample size to strengthen external validity, and conducting additional cycles to assess the sustainability of improvements [8]. Broader application of the three‑tier medication verification checklist across other stress testing modalities may further enhance patient safety and diagnostic reliability [2,17].

## Conclusions

This two‑cycle audit demonstrates that a structured quality improvement approach can enhance the diagnostic accuracy of DSE, with complete elimination of false-positive results following targeted interventions. The introduction of a three‑tier medication verification checklist addressed a preventable source of error and represents a simple, transferable safety intervention applicable across stress testing modalities. These findings highlight the importance of embedding systematic audit processes within routine practice to identify performance gaps, implement corrective measures, and sustain high diagnostic standards. Continued re‑evaluation through future audit cycles will be essential to ensure the long‑term sustainability of these improvements, while multi‑center adoption would strengthen generalizability and demonstrate the broader applicability of this approach to diverse clinical settings.

## Figures and Tables

**Figure 2:**
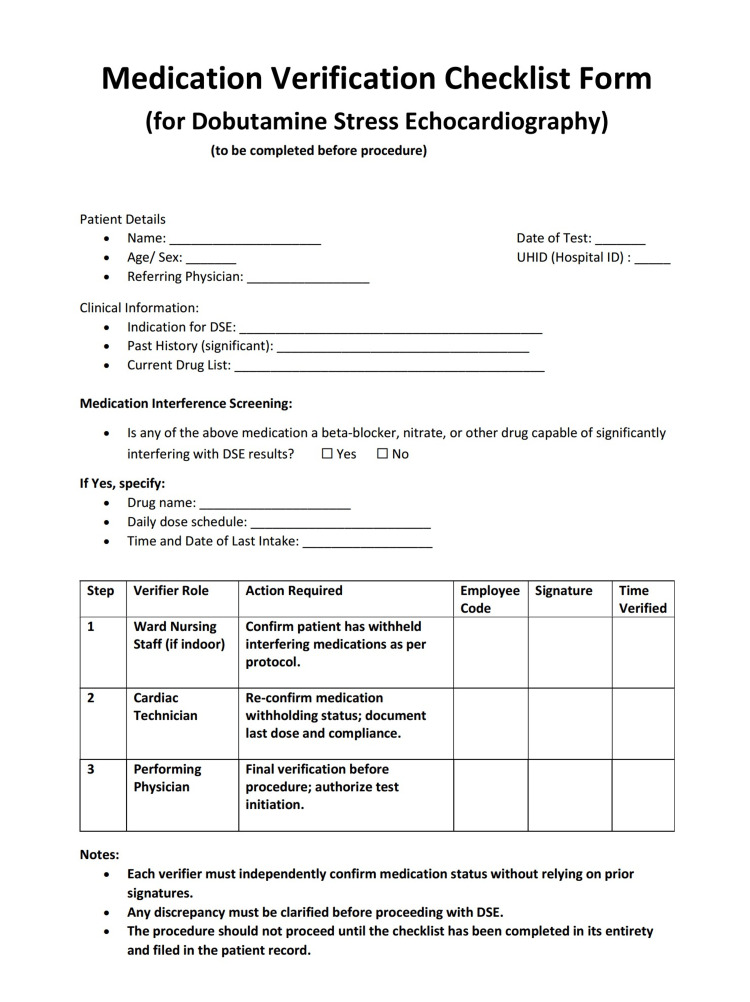
Medication Verification Checklist Form
